# Sema3a inhibits the differentiation of Raw264.7 cells to osteoclasts under 2Gy radiation by reducing inflammation

**DOI:** 10.1371/journal.pone.0200000

**Published:** 2018-07-05

**Authors:** Bo Huang, Qin Zhang, Ying Yuan, Na Xin, Kun He, Yan Huang, Hua Tang, Ping Gong

**Affiliations:** 1 State Key Laboratory of Oral Diseases, West China Hospital of Stomatology, Sichuan University, Chengdu, PR China; 2 Dental Implant Center, West China Hospital of Stomatology, Sichuan University, Chengdu, PR China; 3 OMFS-IMPATH Research Group, Oral Imaging Center, Department of Imaging and Pathology, Biomedical Sciences Group, University of Leuven, Leuven, Belgium; University of Catanzaro, ITALY

## Abstract

Astronauts and cancer patients receive different types of radiation, and radiation decreases bone strength and leads to radiation-induced osteoporosis. This effect is attributed to the activation of osteoclasts. Our aim was to study the effect of Sema3a on the differentiation of the murine macrophage cell line Raw264.7 into osteoclasts upon irradiation. Raw264.7 cells were divided into four groups: A, receiving no radiation; B, receiving no radiation + 50ngng/ml Sema3a; C, receiving 2Gy radiation; and D, receiving 2Gy radiation +50ngng/ml Sema3a. After treatment, cells were subjected to a proliferation assay, migration assay, live and apoptosis assay, and an ROS assay, along with analyses of bone resorption activity, TRAP staining and RT-PCR to assess the effect of Sema3a on Raw264.7 cells under 2Gy radiation. Sema3a inhibited the proliferation of Raw264.7 cells and showed statistical significance at a concentration of 100ngng/ml (P<0.05). Under 2Gy radiation, cell migration was reduced (P<0.05). In addition, 2Gy radiation resulted in more apoptotic cells, a higher level of ROS, larger bone resorption lacunae and more Trap-positive cells (p<0.05), and radiation increased *CSTK*, *NFAT*, *TRAP-5b*, *Rankl/OPG*, *IL-1*, *IL-6*, *TNFa* and *P53* gene expression (P<0.05). Sema3a had an inhibitory effect on the differentiation of Raw264.7 cells and the migration and activity of osteoclasts upon irradiation but did not affect ROS. Sema3a also decreased the expression of *CSTK*, *NFAT*, *TRAP-5b*, *Rankl/OPG*, *IL-1*, *IL-6* and *TNFa* on the 3rd and 7th days after irradiation (p<0.05), whereas *P53* expression was increased (P<0.05). Sema3a reduced the inflammation induced by radiation and negatively regulated osteoclast differentiation. Sema3a promoted Raw264.7 cell apoptosis after irradiation, indicating that Sema3a could be a potential therapeutic target for radiation-induced osteoporosis.

## Introduction

As a clinical therapy to treat cancer, radiation therapy can damage adjacent tissues [1]. Cell cycle arrest, mutagenesis, DNA damage, apoptosis and nucleotide excision repair are induced by radiation and result in cell injury. Radiation also increases the production of reactive oxygen species (ROS) and inflammatory cytokines. ROS suppress cell division, inhibit cell differentiation, and increase cell apoptosis. In addition, persistent DNA-damage signaling associated with cellular senescence is accompanied by the induction of inflammatory factors, including superoxide radicals, hydrogen peroxide, hydroxyl radicals, tumor necrosis factor-α (*TNFα*), and interleukin-1 (*IL-1*) [2–3]. Previous studies have shown that radiation therapy elevates circulating markers of bone resorption and increases the number of osteoclasts in the proximal tibia of mice during the first 3 days after exposure to 2Gy X-ray irradiation [4–7]. Radiation reduces cellular activity, blood supply, and partial oxygen pressure, causing a reduction in bone quality and quantity [7–8]. These unavoidable post-radiation effects may lead to osteopenia and radiation-induced osteoporosis, reductions in bone strength, and an increased risk of serious fractures [4, 6, 7, 9, 10]. Rib fracture rates increase ten-fold in breast cancer patients after radiation therapy. Spaceflight radiation (a special mixture of gamma rays, high-energy protons, and cosmic rays) and low gravity in space are reported to have negative effects on astronauts’ bodies, especially the skeletal system [10–12]. Prolonged exposure to spaceflight radiation, as well as low gravity, results in harm to skeletal health due to an acute increase in the number and activity of osteoclasts [10, 12–13]. The increased osteoclast activity leads to bone mineral density (BMD) loss, which may result in an increased risk of osteoporosis [12,14].

Recent studies demonstrate that Sema3a shows a significant impact on the skeletal system [15–22]. The Sema superfamily, consisting of various glycoproteins, are expressed widely in the human body, including in bone, cartilage, cancer cells, endothelial cells, glia, teeth, neurons, connective tissue and muscles, and are involved in a variety of physiological activities, such as cell apoptosis, cell adhesion, cell migration and patterning, guidance of axonal growth, vascular reconstruction and growth, tumor metastasis, cytokine release and immune cell regulation [23]. Sema3a is a prototype of an axonal guidance molecule in the semaphorin family and contains a conserved extracellular domain of approximately 500 amino acids; Sema3 has been shown to inhibit osteoclast formation and decrease osteoclast activity [15–16,21–22]. Hayashi’s study suggested that Sema3A directly binds to *Nrp-1* and associates with *Plexin-A1*, leading to inhibition of the immunoreceptor tyrosine-based activation motif (ITAM) and RhoA signaling pathways and negatively regulating osteoclast differentiation induced by receptor-mediated activation of the nuclear factor-kB ligand (*Rankl*) pathway [16]. The expression level of *Nrp2* is markedly increased in hematopoietic bone marrow cell-derived osteoclast cultures upon binding class 3 semaphorins. Nevertheless, Nrp2 knockout mice produce more *Rankl* in comparison with wild-type mice, and *Nrp2* knockout mice are characterized by a low bone mass phenotype accompanied by an increased osteoclast count and a decreased osteoblast count [17].

Studies *in vivo* suggest that global knockout of Sema3a results in reduced bone mass and more osteoclasts in mouse femurs [16–17]. In wild-type mice, average bone volume per tissue volume (BV/TV) is 30%, whereas this value is reduced to 8% in the mice with a global knockout of Sema3a (16). Sema3A-/- mice show fusion of cervical bones, partial rib duplication and a poor alignment of the rib-sternum junction. Sema3A also modulates bone vascularization and the recruitment of osteoblasts and osteoclasts during bone development and remodeling (18). Sema3A mitigates bone loss by decreasing osteoclastic bone resorption and increasing osteoblastic bone formation in an ovariectomized mouse model of postmenopausal osteoporosis, and Sema3a suppresses osteoclastogenesis and promotes osteoblastogenesis in cultured cells *in vitro* [16, 24].

Radiation therapy or spaceflight radiation promotes osteoclast differentiation and causes increased bone loss and an elevated risk of osteoporosis, and Sema3a plays an important role in bone remodeling regulation. However, the effect of Sema3a on the differentiation of osteoclast precursor cells to osteoclasts upon irradiation remains unclear [22]. The aim of our study was to examine the effect of Sema3a on Raw264.7 cells subjected to 2Gy gamma radiation, and we predicted that Sema3a would show an inhibitory effect on Raw264.7 cell differentiation and subsequent osteoclast activity.

## Materials and methods

### Cell culture

A clonal population of Raw264.7 cells was cultured in modified Eagle’s medium (DMEM, HyClone, USA) containing 10% fetal bovine serum (FBS, Gibco, Australia) and 1% penicillin-streptomycin (HyClone, USA) and incubated at 37 °C with 5% CO2. The medium was changed every two days.

### Proliferation assay

Upon reaching 80% confluency, the Raw264.7 cells were digested by 0.25% trypsin (HyClone, USA) with ethylene diamine tetraacetic acid (EDTA) and were re-suspended with DMEM. Then, the cells were seeded in 96-well plates at a density of 5X10^4^/ml. After 12 h, the medium was replaced by fresh DMEM containing 2% FBS with or without Sema3a at concentrations of 0, 10, 50, and 100ngng/ml. The viability of the Raw264.7 cells was assessed by a CCK-8 assay (CCK-8, Dojindo, Japan) on days 1, 3, 5, and 7. The optical density (OD) values were measured at 450 nm using a microplate reader (Varioskan Flash; Thermo Fisher Scientific, USA). The 50ngng/ml group showed the best result in the proliferation assay and thus was used in the subsequent experiments.

### Radiation

The cells were digested with 0.25% trypsin and re-suspended in DMEM containing 2% FBS. Then, the cells were divided into four groups: Group A, 0Gy radiation; Group B, 0Gy radiation + 50ngng/ml Sema3a; Group C, 2Gy radiation; and Group D, 2Gy radiation + 50ng/ml Sema3a. A single dose of 2Gy gamma radiation was administered at a rate of 0.83 Gy/min using a linear accelerator at the Seventh People’s Hospital of Chengdu, China.

### TRAP staining

After the radiation treatment, the cells were cultured in the presence of 50ng/ml of Rankl to induce osteoclast formation. After 7 days, the cells were fixed with paraformaldehyde and stained following the manufacturer’s instructions. TRAP-positive cells containing more than three nuclei were identified, the nuclei were counted, and images were captured using an inverted phase contrast microscope (LEICA ZE4 HD, high definition, Germany).

### Cell migration assay

The cell migration assay was performed using 24-well-transwell plates (Corning Corp; USA) according to the manufacturer’s instructions. In this experiment, the Raw264.7 cells given 2Gy or 0Gy radiation were plated in the upper chamber with no FBS, and medium with 10% FBS containing 0 or 50ng/ml Sema3a was added to the bottom chamber. Then, the cells were cultured in the incubator at 37 °C with 5% CO_2_; 24 and 72 h later, the cells on the upper side of the membrane were scrubbed gently with a cotton-tipped swab. The migratory cells on the lower surface of the membrane were fixed with 95% methanol and were stained with 0.1% crystal violet (Sigma-Aldrich, MO, USA). The stained migratory cells were photographed under an inverted light microscope and were quantified by manually counting five randomly selected areas of view. Five independent experiments were performed.

### Bone resorption activity analysis

The ability of osteoclasts to form resorption lacunae on bovine cortical bone slices (0.5 mm in thickness and 5 mm in diameter) was tested. The cells were plated at a concentration of 5X10^4^ cells in 48-well culture plates containing bovine cortical bone slices. To support osteoclast differentiation, the cells were cultured in complete DMEM medium supplemented with 10% FBS, 1% penicillin/ streptomycin and M-CSF (30ng/ml) for the first two days. Then, Rankl (50ng/ml) was added to the culture medium from the third day to the end of the experiment. The cell culture medium was replaced every two days. The cells were incubated for 10 days to form resorption lacunae. Bovine cortical bone slices were ultrasonically cleaned three times for 5 min each in ultrapure water to remove the cells; the bone slices were then rinsed in PBS and naturally dried. Finally, a scanning electron microscope (SEM; HITACHI S3400+EDX, KEKY 2800, Germany) was used to visualize lacunar resorption.

### Cell apoptosis assay

A cell apoptosis assay was performed using the Annexin V-FITC/PI apoptosis detection kit (KeyGEN, Nanjing, China). The cells were seeded in 6-well plates (1.0 × 10^5^ cells/well) followed by treatment with 2Gy radiation and/or Sema3a, as described above, for 24 h. The untreated cells were used as the control group. At the end of the incubation period, the cells were harvested and washed with PBS twice and incubated in a dark room with Annexin V-FITC and propidium iodide for 15 min; then, the stained cells were analyzed using a flow cytometer (Beckman Cytomic FC500,USA).

### ROS assay

ROS levels were analyzed in the four groups at 2 and 8 h after treatment, following the manufacturers’ instructions. Intracellular ROS was marked with DCFH-DA (Sigma, USA), and the cell nuclei were labeled with DAPI (Sigma, USA). The cells were then washed 3 times with PBS. The cell culture samples were examined with a fluorescence microscope (Zeiss Axioplan; 10×40) equipped with a FITC and a DAPI filter.

### Real-time PCR assay and western blot analysis

On the third day and seventh day after treatment, total RNA was extracted with an RNA extraction kit (Bioer Technology, China) following the manufacturer’s protocol. The concentration of the RNA was measured by a spectrophotometer, and the samples with an OD value (A260/A280) between 1.8 and 2.0 were reverse transcribed into cDNA using the Prime Script TM RT-PCR kit (Takara, Japan). The cDNA products were amplified using Takara Taq^™^ (DR001AM, Takara, Japan) for 40 cycles (denaturation for 30 s at 95 °C, followed by primer annealing for 5 s at 95 °C and an extension for 31 s at 60 °C). Each real-time PCR was carried out in quadruplicate using the ABIPRISM 7300 real-time PCR system (Applied Biosystems, US). Levels of NFAT, CTSK, Trap-5B, Rankl/Opg, IL-1, IL-6, TNFa and P53 were examined, and GAPDH was used as an internal control. The sequences of the primer pairs used are provided in Table 1. At 3 days, whole-cell protein was obtained using lysis buffer (Keygen total protein extraction kit, Keygen Biotech, China). After boiling for 5 min, 50 mg of protein was subjected to SDS-PAGE (10% polyacrylamide gel) at 60 V for 60 min and 100 V for 80 min. After transfer, membranes were incubated with the relevant antibody (1:2000), and reactive bands were visualized using an enhanced chemiluminescence (ECL) kit (Millipore, Billerica, MA). The results were analyzed by a densitometer (Quantity One, Bio-Rad).

**Table 1:** The primer pairs were used in the study.

Gene	Primer forward	Primer reverse
GAPDH	5’-AGAACAGAGTCATCCCACAC-3’	5’-GCTACGTTATTCTTGCCATC-3’
IL-6	5’-CCAATTTCCAATGCTCTCCT-3’	5’-ACCACAGTGAGGAATGTCCA-3’
P53	5’-ACCATCATCACGCTGGAAGACT-3’	5’-CTGGTGGGCAGTGCTCTCTT-3’
IL-1	5’-GCTCTGCCATTGACCATCTTTC-3’	5’-CTGTTACTGCCACCACATTCTCC-3’
CTSK	5’-CAGCTTCCCCAAGATGTGAT-3’	5’-AGCACCAACGAGAGGAGAAA-3’
NFAT	5’-CCCCACGCCTTCTATCA-3’	5’-GTCGGTCTCGCCTTTCC-3’
Rankl	5’-CCTGAGGCCCAGCCATTT-3’	5’-CTTGGCCCAGCCTCGAT-3’
OPG	5’-TCCTGGCACCTACCTAAAACAGC-3’	5’-CTACACTCTCGGCATTCACTTTGG-3’
Trap5b	5’-CAGCAGCCAAGGAGGACTAC-3’	5’-ACATAGCCCACACCGTTCTC-3’
TNF-a	5’-GACATCACTGGAGTTTCCCCT-3’	5’-CCCTCCATACACCCGACTTT-3’

### Statistical analysis

All data were analyzed with SPSS 21.0, using a one-way analysis of variation (ANOVA), followed by the Student-Newman-Keuls test for multiple comparisons. All data are expressed as the mean ± SEM. A P value < 0.05 was considered statistically significant. Four to five independent replicates for each experiment were conducted.

## Results

### Sema3a inhibits the proliferation of Raw264.7 cells

**Fig 1:**
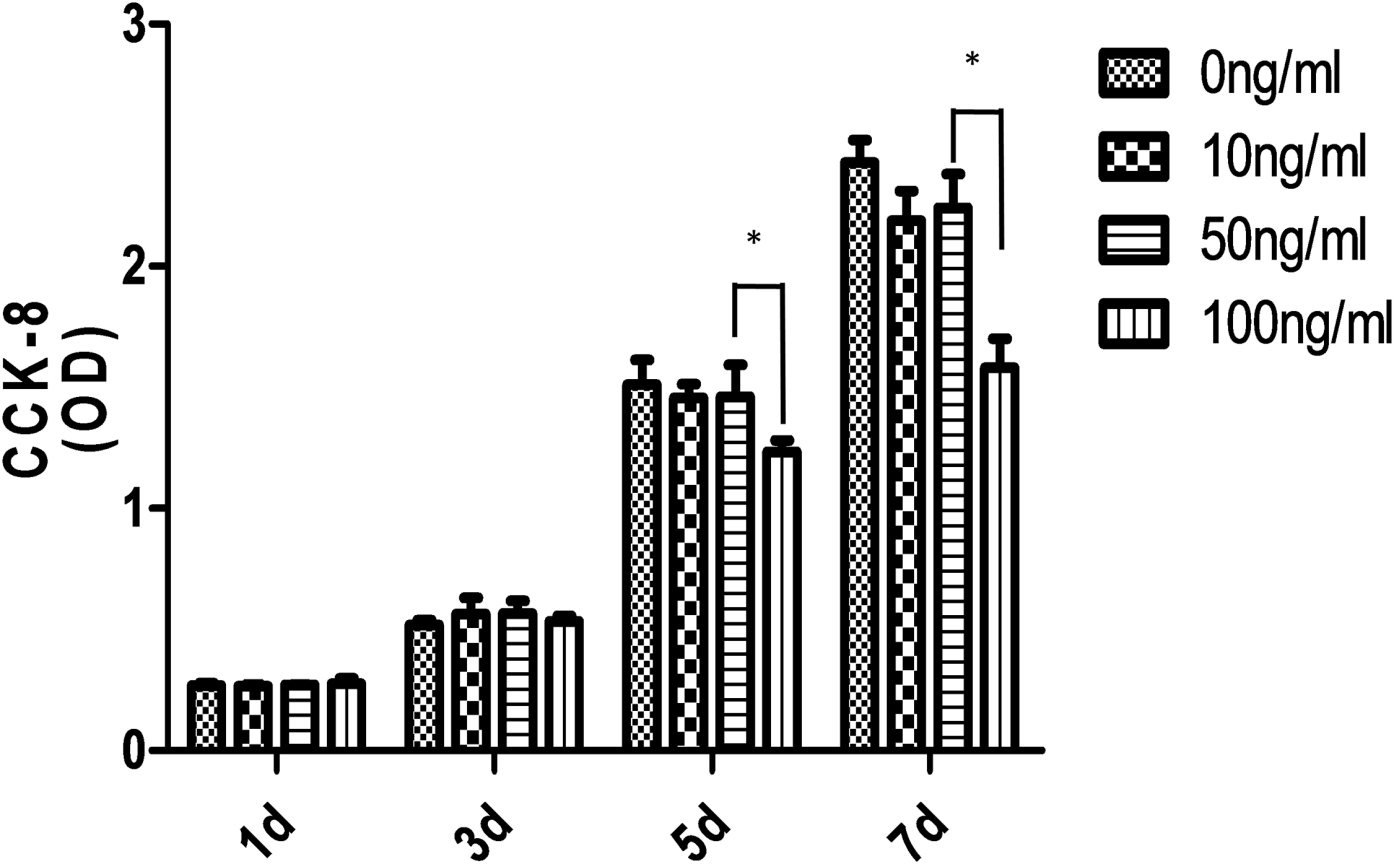
Cell proliferation. Sema3a at concentrations of 0ng/ml, 10ng/ml, 50ng/ml and 100ng/ml was used to analyze the effect on cell proliferation. On days 5 and 7, 100ng/ml significantly reduced cell proliferation compared to 0ng/ml (*P<0.05, N = 5).

To screen for the proper concentration of Sema3, we initially examined the effects of different concentrations of Sema3a on the proliferation of the Raw264.7 cells using a CCK-8 assay kit. As shown in Fig 1 (The raw data could be seen in S1 Table), on the 5th and 7th days, 10ng/ml, 50ng/ml and 100ng/ml Sema3a inhibited cell proliferation; 10ng/ml and 50ng/ml of Sema3a did not reach statistical significance (P>0.5), but 100ng/ml of Sema3a showed a significant inhibition of cell proliferation (P<0.05). Because 50ng/ml of Sema3a showed no significant effect on cell proliferation, this concentration was used in the subsequent experiments.

### Sema3a inhibits Raw264.7 cell differentiation into osteoclasts

**Fig 2:**
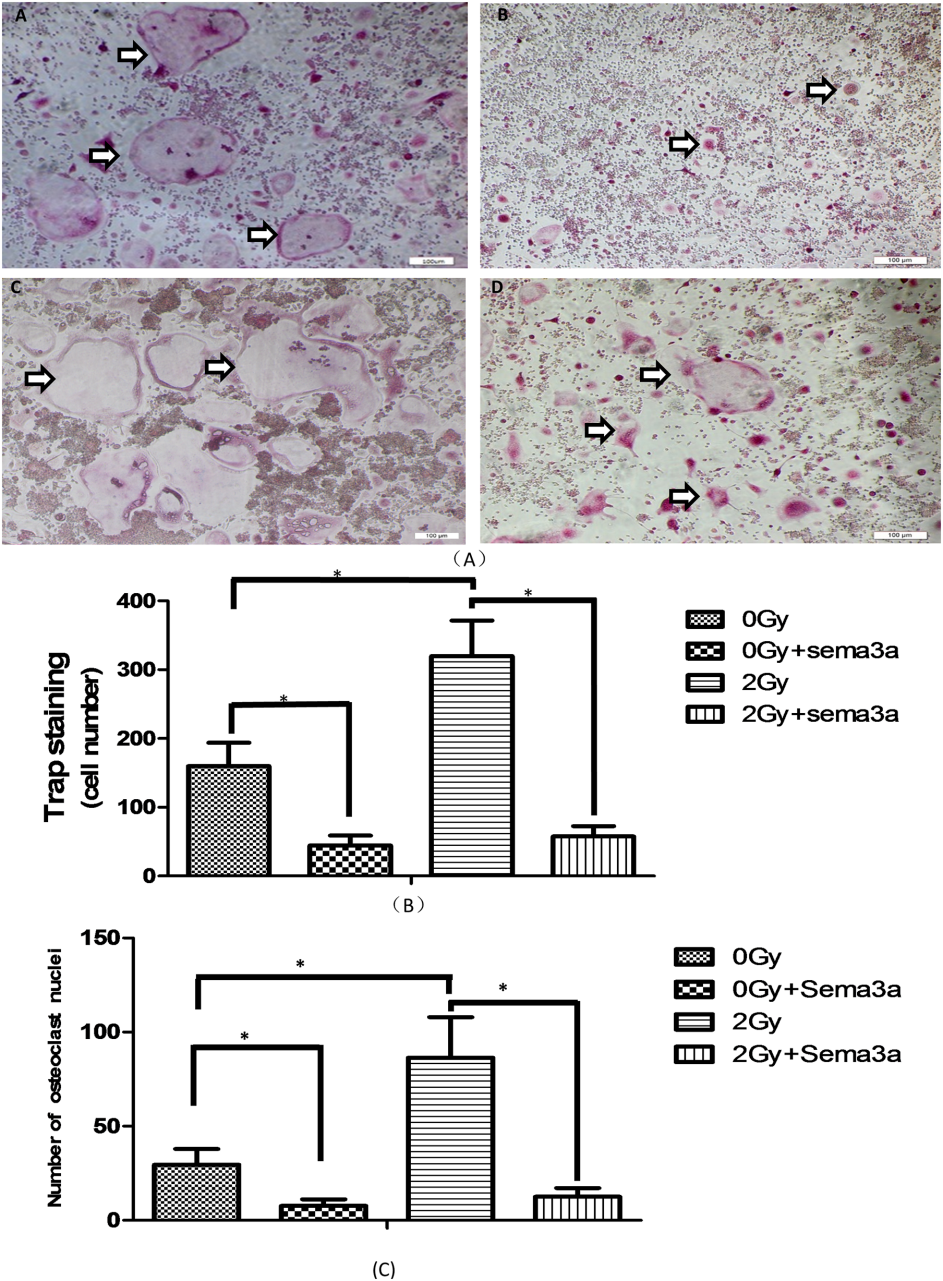
Osteoclast differentiation. 2A shows the Trap staining: After addition of Rankl to the four groups for 7 days, the cells were fixed and stained for Trap. The osteoclasts were stained red. The white-black arrows indicate the osteoclasts. A: 0Gy + 0ng/ml Sema3a; B: 0Gy + 50ng/ml Sema3a; C: 2Gy + 0ng/ml Sema3a; D: 2Gy + 50ng/ml Sema3a. A greater number of positive cells with more nuclei were seen in groups A and C. Fig. 2B shows the number of osteoclasts: Sema3a apparently inhibited osteoclast differentiation under 0Gy radiation and 2Gy radiation, and 2Gy radiation significantly promoted osteoclast differentiation without Sema3a (*P<0.05). With 50ng/ml of Sema3, the differences between 0Gy radiation and 2Gy radiation were not statistically significant (N = 5). Fig. 2C shows the number of osteoclast nuclei (P<0.05, N = 6).

TRAP staining was used to analyze the formation of osteoclasts. As shown in Fig 2A, a greater number of TRAP-positive multinucleated cells that also showed larger cell size were observed after 2Gy radiation compared with 0Gy radiation (P<0.05). The number of TRAP-positive multinucleated cells significantly decreased with 50ng/ml Sema3a compared with no Sema3a under 2Gy and 0Gy radiation (P<0.01) (Fig 2B), with similar results for nuclei number, indicating that Sema3a had an inhibitory effect on osteoclast formation (Fig 2C) (The raw data could be seen in S2 Table).

### Effects of radiation and Sema3a on osteoclast bone resorption activity

**Fig 3:**
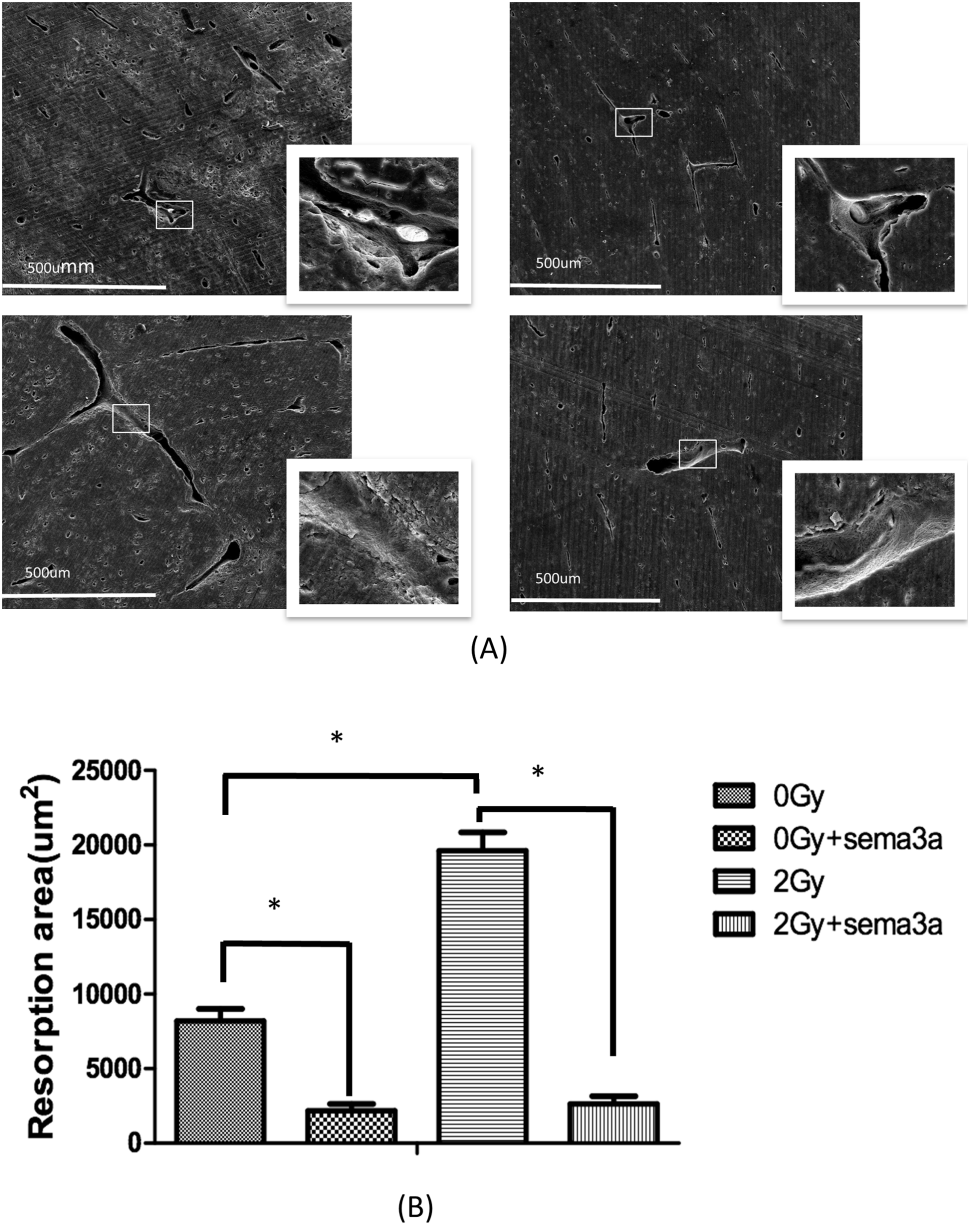
Bone resorption. 3A shows the results of the bone resorption assay: Raw264.7 cells plated on bovine cortical bone slices were differentiated into osteoclasts by the addition of 50ng/ml of Rankl and 20ng/ml of M-CSF in the presence or absence of 50ng/ml Sema3a and/or 2Gy radiation. The cells were then lysed with 5% sodium hypochlorite for 15 min, and the bone slices were subsequently dried. The resorbed areas were photographed by SEM and were analyzed by the ImageJ program. Scale bars = 500 μm (N = 5). Fig. 3B shows the area of bone resorption assay: The resorbed areas were measured with LSM Image Browser software. The area of the bone resorption increased two-fold upon 2 Gy radiation compared to 0 Gy radiation; with Sema3a, bone resorption area decreased 4-fold and 6-fold in the 0 Gy and 2 Gy groups, respectively. (*P<0.05, N = 6).

Bovine cortical bone slices were used to test the osteoclast activity. As Fig 3A shows (The raw data could be seen in S3 Table), the area of lacunar resorption was significantly increased in the 2Gy radiation group compared with the 0Gy radiation group (P<0.01). Compared to the group treated with 2Gy radiation alone, the area of lacunar resorption was significantly decreased when the cells were treated with both Sema3a and 2Gy radiation (P<0.01). With 50ng/ml Sema3, the difference between 2Gy and 0Gy was not statistically significant (Fig 3B).

### The effect of Sema3a on cell migration under 2Gy radiation

**Fig 4:**
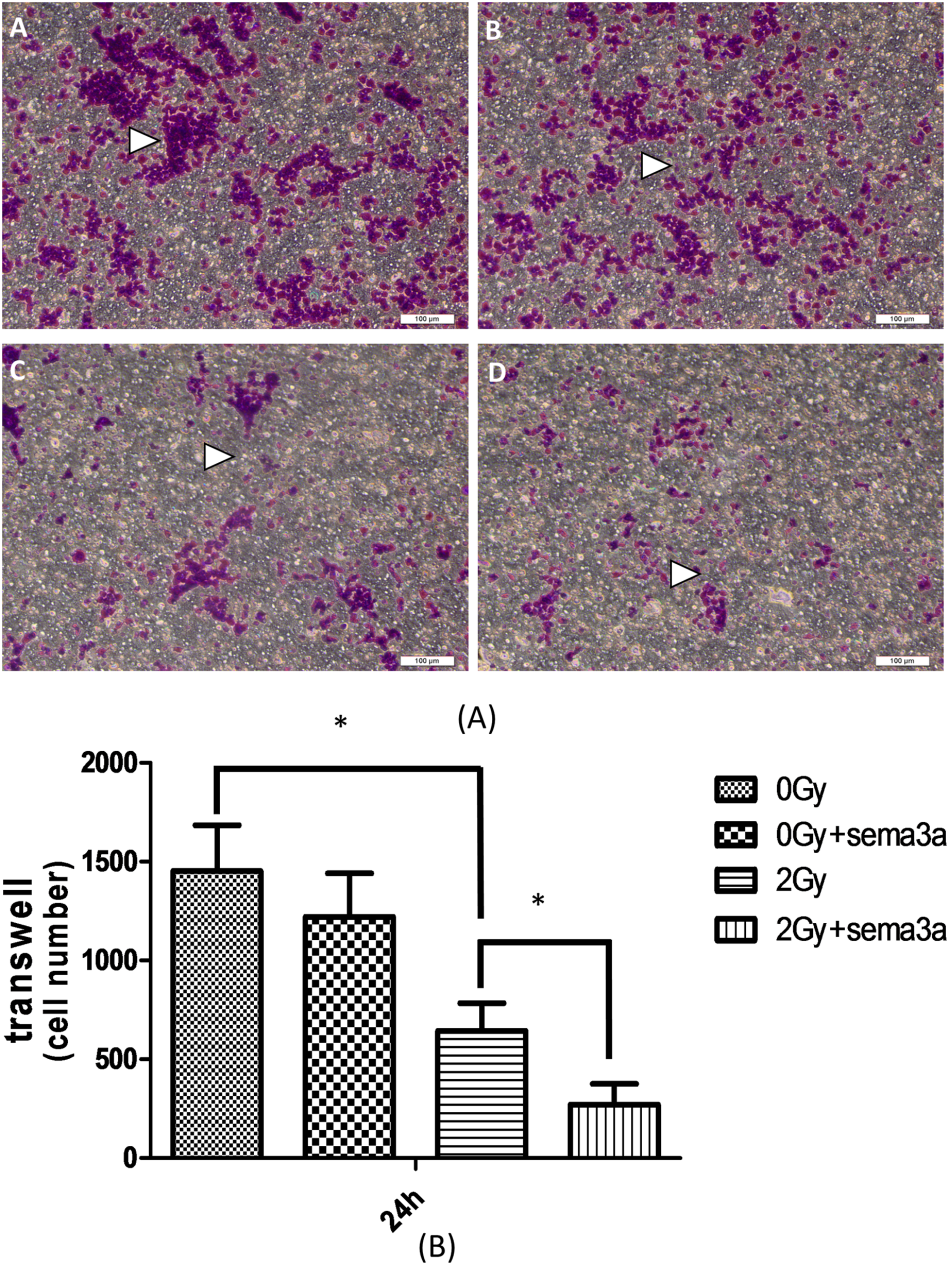
Cell migration assay. Raw264.7 cells given 2Gy or 0Gy radiation were placed in the upper chamber with no FBS, and medium with 10% FBS containing 0 or 50ng/ml Sema3a was added to the bottom chamber; 24 h later, cells were fixed with methanol and stained with crystal violet for 10 min. The cells on the upper side of the membrane were scrubbed away gently. The white triangles indicate the cells that migrated from the upper chamber to the lower chamber (Fig. 4A). A: 0Gy + 0ng/ml Sema3a; B: 0Gy + 50ng/ml Sema3a; C: 2Gy + 0ng/ml Sema3a; D: 2Gy + 50ng/ml Sema3a (N = 5). Fig. 4B shows the number of migrated cells: 2Gy radiation significantly decreased cell migration (*P<0.05), and Sema3a also inhibited cell migration; however, only under 2Gy radiation did Sema3 show a statistically significant effect (*P<0.05, N = 5).

As shown in Fig 4a and 4b, more cells migrated to the lower surface of the transwell membrane after 2Gy radiation compared with the un-irradiated cells (P<0.05). Under 2Gy radiation, 50ng/ml of Sema3 resulted in fewer cells on the lower surface of the transwell membrane compared to 0ng/ml of Sema3a (p<0.05). In contrast, no statistically significant difference was detected between 2Gy radiation and 0Gy radiation with 50ng/ml of Sema3a (The raw data could be seen in S4 Table).

### Sema3a and 2Gy radiation stimulates cell apoptosis

**Fig 5:**
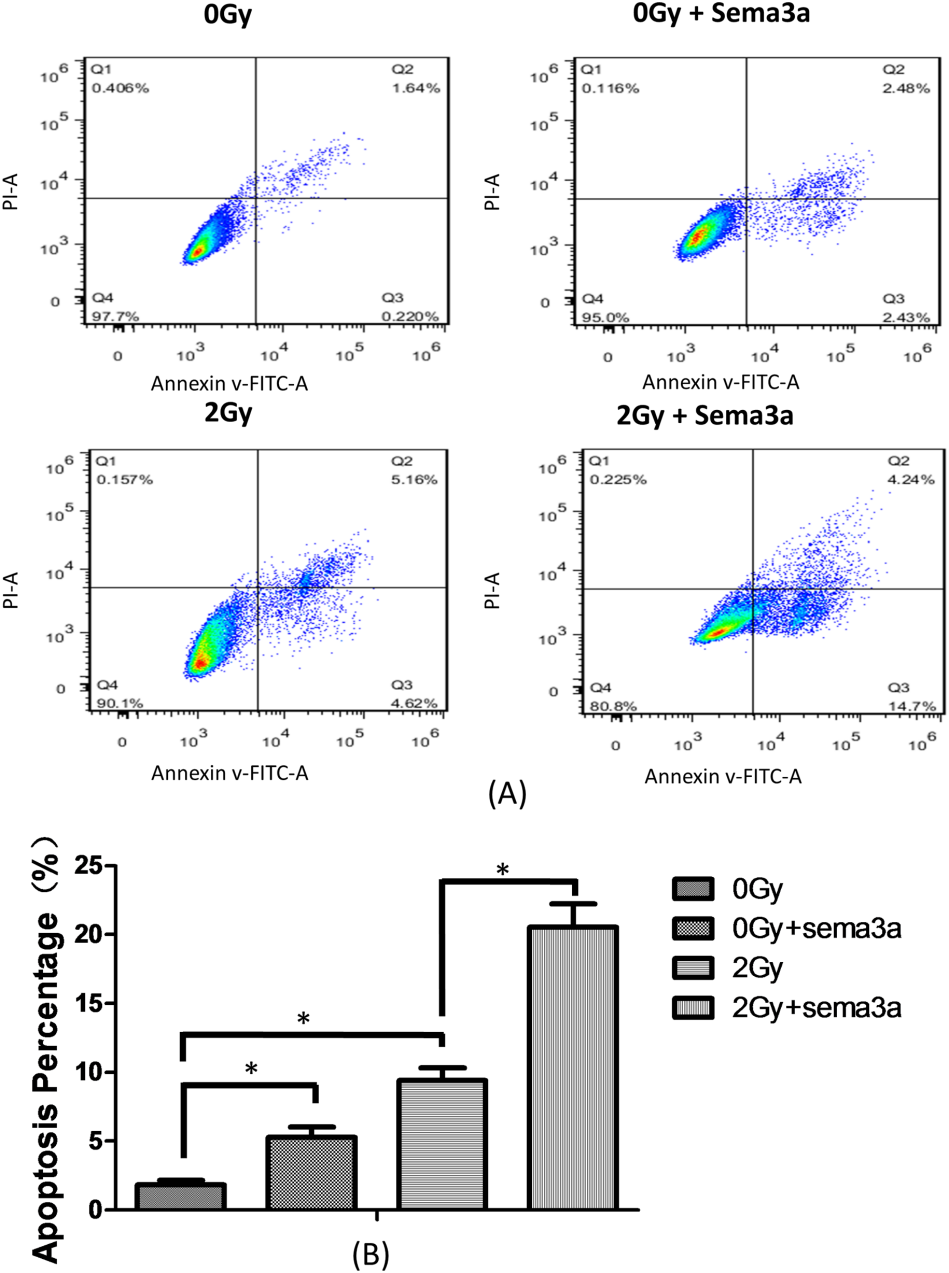
Cell apoptosis assay. The cells were divided into four groups as described before (A: 0Gy + 0ng/ml Sema3a; B: 0Gy + 50ng/ml Sema3a; C: 2Gy + 0ng/ml Sema3a; D: 2Gy + 50ng/ml Sema3a). (A) Annexin V-FITC/PI double staining by flow cytometry. (B) Statistical analysis of the cell apoptosis assay. The number of apoptotic cells was the sum of Q2 and Q3 (P<0.05, N = 4). (Q1, PI positive and annexin negative; Q2, both annexin and PI positive; Q3, annexin positive and PI negative Q4, both annexin and PI negative).

To quantitatively analyze the extent of apoptosis caused by radiation and Sema3a, an Annexin V/ PI staining assay was performed (Fig 5) (The raw data could be seen in S5 Table). The percentages of viable cells (Q4) for 0Gy, 2Gy, 0Gy + Sema3a and 2Gy + Sema3a were 97.7%, 90.1%, 95% and 80.8%, respectively (Fig 5a). The percentages of apoptotic cells (Q2+Q3) in the 0Gy, 2Gy, 0Gy + Sema3a and 2Gy + Sema3a groups were 1.86%, 9.78%, 4.91% and 18.94%, respectively (Fig 5B, P<0.05), while the percentage of necrotic/dead cells (Q1) was similar among the four groups. The apoptosis results are quantified in Fig 5B.

### The effect of radiation and Sema3a on ROS generation in Raw264.7 cells

**Fig 6:**
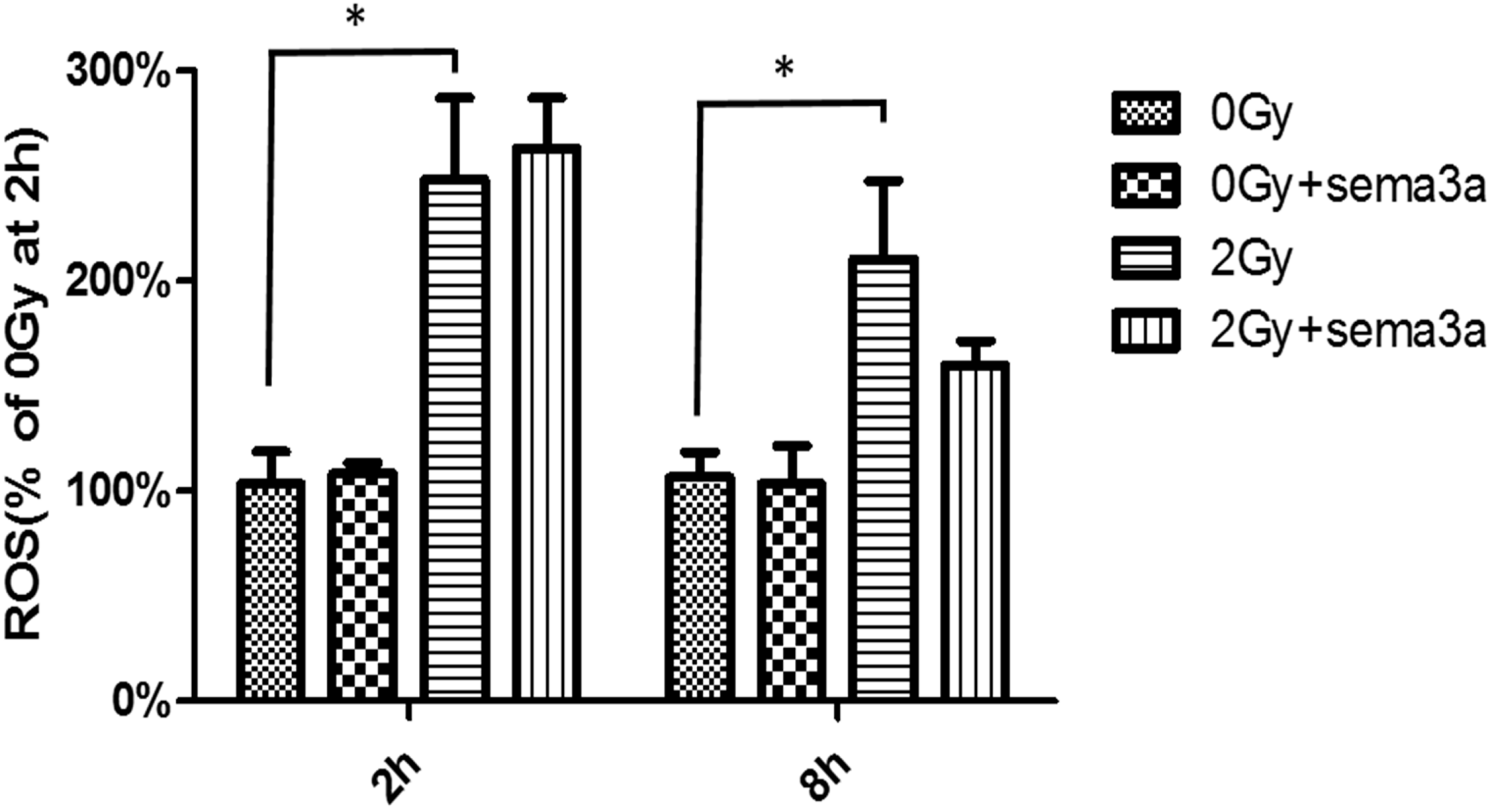
ROS levels. At 2 h, the ROS level increased approximately 4-fold upon 2Gy radiation compared to 0Gy radiation (*P<0.05), and Sema3a showed no effect on ROS generation under 0 Gy or 2Gy radiation. At 8 h following 2Gy radiation, the ROS level decreased approximately 10% compared to 2 h. Although Sema3a also decreased the ROS level, the differences between the 0ng/ml of Sema3a group and the 50ng/ml Sema3a group were not statistically significant (P>0.05, N = 5).

The DCFH-DA fluorescence probe was used to measure the effect of 2Gy radiation and Sema3a on ROS generation in the Raw264.7 cells (Fig 6) (The raw data could be seen in S6 Table). Two hours after irradiation, the level of ROS increased approximately 2-fold with 2Gy radiation compared to 0Gy radiation (p<0.05). Sema3a showed no effect ROS generation in either the 0Gy and 2Gy radiation groups. At 8 h, 2Gy radiation was associated with approximately 10% reduction in ROS levels compared to the 2 h time point. Although Sema3a also decreased the ROS level at 8 h, the differences between the 2Gy radiation group and the 2Gy radiation + 50ng/ml Sema3a group showed no statistical significance (P>0.05).

### Real-time RT-PCR and western blot analysis

**Fig 7:**
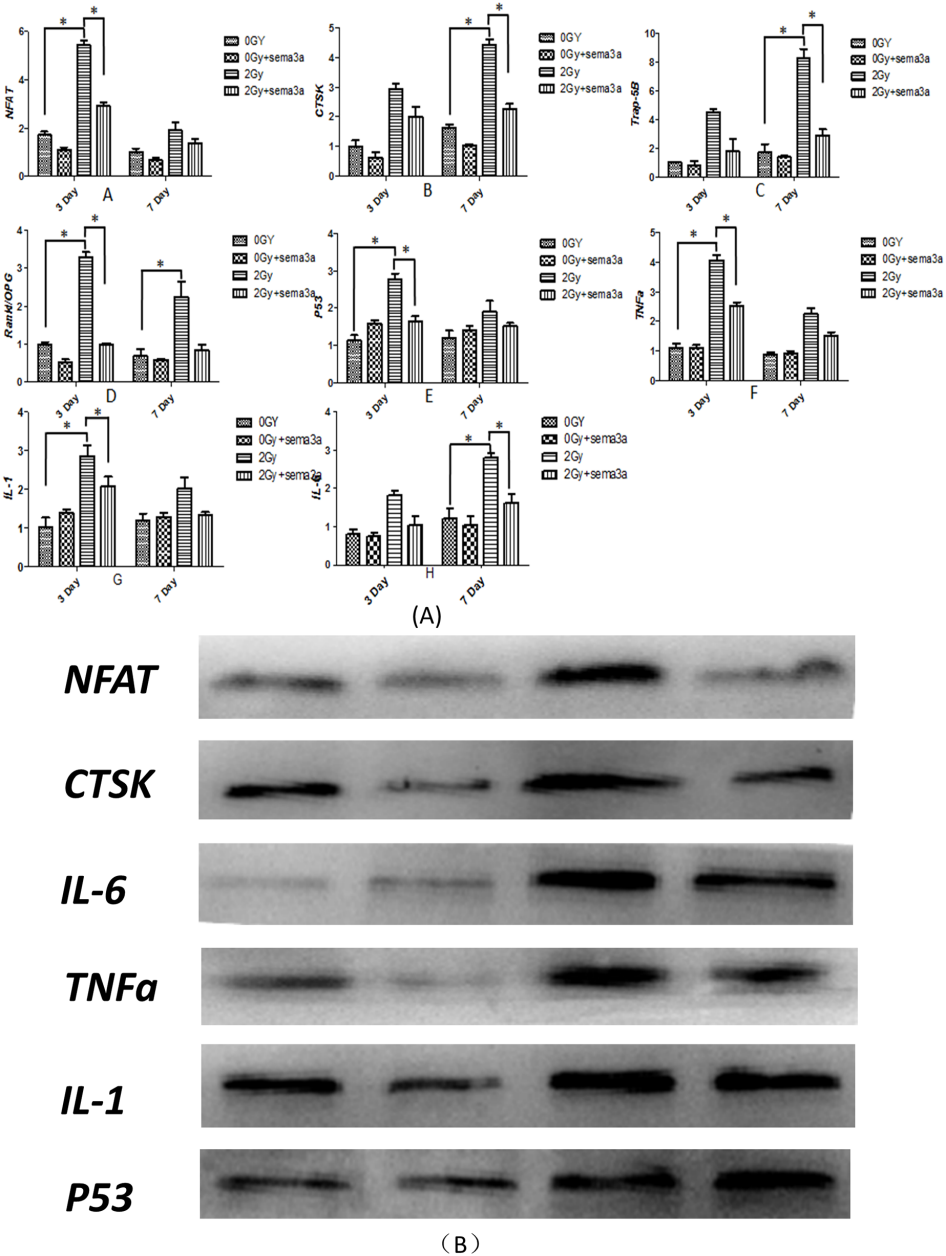
Gene expression. The expression levels of *NFAT*, *CTSK*, *TRAP-5b*, *Rankl/OPG*, *IL-6*, *TNFa*, *IL-1* and *P53* on the 3rd and 7th days are shown (Fig. 7A). Gene expression levels of NFAT and *Rankl/OGP* were higher on the 3rd day than on the 7th day, and 2Gy radiation significantly increased their expression at both time points. Sema3a decreased the expression of these genes in the 2Gy and 0Gy radiation groups. The expression of *CTSK* and *TRAP-5b* was higher under 2Gy radiation compared to 0Gy radiation at both time points, and Sema3a significantly reduced their expression at both time points. The expression of pro-inflammatory genes (*IL-1*, *IL-6 and TNFa*) increased with 2Gy radiation compared with 0Gy. The expression of the *IL-6* and *TNFa* genes was higher on the third day than on the 7th day, but expression of the *IL-1* gene was higher only on day 7. Sema3a significantly decreased *IL-1*, *IL-6*, *and TNFa* expression in the 2Gy group at both time points. With 2Gy radiation, the expression of the apoptosis-related gene P53 was higher on the 3rd day than on the 7th day. Sema3a increased *P53* expression at both time points. (*P<0.05, N = 5). Western blot analysis of *CTSK*, *NFAT*, *IL-1*, *IL-6*, *TNFa* and *P53* levels at 3 days post-radiation is shown in Fig 7B. The results indicate that 2Gy radiation increased levels of *CTSK*, *NFAT*, *P53*, *IL-1*, *IL-6* and *TNFa*. Sema3a showed a similar effect on the expression of these proteins, with the exception of *P53*, in both the 0Gy and 2Gy groups. (N = 5).

RNA levels of *IL-1*, *IL-6*, *TNFa*, *CTSK*, *NFAT*, *TRAP-5b*, *Rankl/OPG* and *P53* were analyzed at the 3rd and 7th days post-irradiation, and the results are shown in Fig 7A (The raw data could be seen in S7 Table). The expression of pro-inflammatory genes, including *IL-1*, *IL-6* and *TNFa*, significantly increased upon 2Gy radiation compared to the non-radiated group at both time points (p<0.05). Gene expression of IL-1 and *TNFa* was higher on day 3 day than day 7, whereas the expression of IL-6 was higher on day 7. Sema3a caused a decrease in the expression of *IL-1*, *IL-6* and *TNFa* in the Raw264.7 cells under 2Gy radiation (P<0.05) at both time points. The expression of the *CTSK* gene was higher on the 7th day than on the 3rd day under 2Gy radiation (P<0.05). However, its expression significantly decreased with the addition of 50ng/ml of Sema3a compared to 0ng/ml of Sema3a (P<0.05). The expression of an osteoclast-related transcription factor gene (*NFAT*) increased approximately 3-fold and 1.5-fold under 2Gy radiation on the 3rd day and the 7th day compared to 0Gy radiation, respectively. In contrast, Sema3a reduced *NFAT* expression by 50% and 20% on the 3rd and 7th days, respectively, after 2Gy radiation. Tartrate-resistant acid phosphatase 5b (*TRAP-5b*), a biomarker for osteoclast-mediated bone resorption, was dramatically upregulated upon 2Gy radiation with osteoclast differentiation compared to 0Gy radiation. *TRAP-5b* expression was reduced 2-fold under 2Gy radiation with 50ng/ml of Sema3a compared to 0ng/ml of Sema3a on the 3rd day and 3-fold on the 7th day. With 0Gy radiation, 50ng/ml Sema3a also decreased *TRAP-5b* expression, but the difference did not reach statistical significance. On the 7th day, *TRAP-5b* expression was obviously higher than on the 3rd day. The apoptosis-related gene *P53* was also analyzed; 2Gy radiation increased *P53* expression at the 3rd and 7th days compared to 0Gy radiation (P<0.05). Sema3a also increased P53 expression in the 2Gy and 0Gy radiation groups on the 3rd and 7th days. However, only on the 3rd day in the 2 GY radiation group did 50ng/ml of Sema3a show a statistically significant effect (P<0.05).

We then examined the protein expression of these markers 3 days after the stimulus. As shown in Fig 7B, 2Gy radiation increased the expression of osteoclast-related proteins and inflammation-related proteins, including *CTSK*, *NFAT*, *TNFa*, *IL-1*, *IL-6* and *P53*, compared with the un-irradiated groups. In contrast, Sema3a showed an inhibitory effect on the expression of these proteins, with the exception of *P53*, in both the 0Gy radiation group and the 2Gy radiation group.

## Discussion

Clinical studies have demonstrated that patients with cancer receive approximately twenty to thirty 2Gy fractions of radiation over 6 weeks, for a total dose of approximately 50Gy. Astronauts in space are subjected to microgravity and space radiation for an extended period of time, and studies show that space radiation induces bone loss in astronauts and increases osteoclast number and activity [10,11,14,25]. A deterioration in bone quantity and quality, a reduction in bone strength leading to mission-critical fractures, and an increased risk of radiation-induced osteoporosis are found in cancer patients given radiation therapy and in astronauts [10, 11, 14, 26–27]. An increase in the area of resorption surfaces lining the trabeculae and the near-complete suppression of bone formation are observed within 2 weeks after irradiation [5, 7, 28–29]. In our study, exposure of Raw264.7 cells to 2Gy gamma radiation and treatment with Rankl led to a dramatic increase in the number of TRAP-positive multinucleated cells, and the area of lacunar resorption in bovine cortical bone slices was elevated compared with the no radiation group. This result indicates that 2Gy radiation increased the number and activity of osteoclasts, consistent with previous *in vivo* studies showing a significant increase in the number and activity of osteoclasts within the first three days after irradiation [5, 7, 28–29]. In our study, a greater number of osteoclast nuclei were observed, and the size of the osteoclasts was increased in the irradiated groups (Fig 2). However, *in vivo* studies showed that TRAP-positive cells with few nuclei or a single nucleus are present after irradiation [5, 29], perhaps because gamma radiation has a negative effect on osteoclast maturation *in vivo* by inhibiting the generation of osteoblastic cells derived from osteoclast-stimulating factors [5, 29]. However, this effect was not observed *in vitro*.

Radiation induces the production of various forms of ROS and numerous inflammatory cytokines [2–3]. In our study, ROS levels dramatically increased after the Raw264.7 cells received 2Gy radiation, followed by a reduction in ROS levels over time. Radiation initially causes the ionization and excitation of water to form water radiolysis products, such as hydrated electrons, ionized water, hydroxyl radicals, hydrogen radicals, and hydrogen peroxide, in a very short period of time; these products are called reactive oxygen species [3]. ROS lead to an increase in osteoclast formation through the activation of Rankl, a key molecule that causes osteoclast differentiation and, maturation. Rankl regulates the *JNK*, *AKT*, *NF-kB*, *ERK* and *p38* signaling pathways to control osteoclast differentiation [30–32]. In addition to ROS, inflammatory cytokines such as *TNFα*, *IL-1*, and *IL-6* were notably higher after 2Gy radiation compared to the un-irradiated group in our study. On the 3rd day after irradiation, the mRNA levels of *TNFα* and *IL-1* were higher than on the 7th day, but the *IL-6* level was showed an additional increase on day 7 compared with day 3. Previous studies have shown that inflammation is a common response to radiation exposure, and radiation upregulates expression of inflammatory cytokines [21, 33]. Jeffrey suggested that radiation in space and radiation therapy affect normal tissues, such as bone and bone marrow [10]. After the maturation and apoptosis of rapidly dividing cells caused by atom ionization or ROS production, macrophages and neutrophils are recruited to remove the apoptotic cells. Macrophage activation and infiltration lead to further increased pro-inflammatory cytokine levels. *TNFα* and *IL-1* directly activate osteoclasts and induce the expression of *Rankl* in stromal cells. These effects may cause rapid bone loss, which leads to an increased lifetime risk of fracture [10].

Sema3a is reported to play an important role in bone metabolism, which mitigates bone loss by both inhibiting osteoclastic bone resorption and promoting osteoblastic bone formation [15–16, 21, 26]. The present study showed that Raw264.7 cells treated with Sema3a resulted in fewer TRAP-positive cells and smaller bone resorption lacunae. These data suggest that Sema3a had an inhibitory effect on the differentiation of Raw264.7 cells into osteoclasts, and Sema3a reduced osteoclast activity. In the 2Gy radiation group, Sema3a showed the same effects. On the 3rd and 7th days after irradiation, gene expression analysis of the four groups showed that 2Gy radiation clearly elevated the expression levels of osteoclastogenic signaling molecules, including *CTSK*, *NFAT*, *TRAP-5b*, *and Rankl/OPG*, *NFAT* and *Rankl/OPG;* these factors were higher on day 3 than day 7, whereas *CTSK* and *TRAP-5b* were higher on the 7th day, similar to the study by Joshua S’ [34]. Upon treatment with Sema3a, these osteoclastogenic signaling molecules were significantly decreased, suggesting that Sema3a negatively regulated osteoclastogenic gene expression and mitigated the effect of radiation on osteoclast differentiation. The effects of Rankl are inhibited by the binding of Sema3a to neuropilin-1 (*Nrp1*), with accompanying impairment of the immunoreceptor tyrosine-based activation motif (ITAM) and RhoA signaling pathways (16). Therefore, Sema3a may inhibit osteoclast differentiation by repressing the *NF-kB* pathway activated by radiation.

During macrophage differentiation and T cell activation, the expression of Sema3a increases, indicating that Sema3a plays a vital role in modulating inflammatory conditions and implicating it as a potent immunoregulator during all immune response stages [35–36]. In retinopathy models, mRNA expression of the inflammatory cytokines *IL-1β* and *TNFα*, as well as Sema3a, increased significantly compared to controls [37]. The same study also showed that *IL-1β*, produced by activated retinal microglia, induced the adjacent neurons to release Sema3a, which was significantly attenuated by inhibitors of *IL-1R* [37]. Tanaka and Alfonso demonstrated that Sema3a suppressed the release of Th2-related cytokines (*IL-5*, *IL-13 and IL-4*) and inflammatory cytokines (I*FN-α*, *IL-17 and TNF-α*) but increased levels of the cytokine *IL-10* in experimental allergic conjunctivitis and autoimmune arthritis models [38–39]. Although Sakai demonstrated that co-stimulation with *IL-4* and *TNF-α* increased epidermal innervation and was accompanied by the suppression of Sema3a, the mechanism of Sema3a regulation under inflammatory conditions has not yet been elucidated [40]. In our radiation model, Sema3a caused a decrease in mRNA expression of the inflammatory cytokines *IL-1β*, *IL-6* and *TNF-a*, suggesting that Sema3a regulated the inflammation caused by radiation. Inflammatory cytokines induced by radiation cause increased expression of *Rankl* in bone marrow cells or osteoblasts, which then activates the *NF-KB* pathway to increase osteoclast differentiation and activity [5, 7, 10, 29]. Sema3a may suppress osteoclast formation by downregulating the inflammatory cytokines induced by radiation.

In this study, we also observed a suppressive effect of Sema3a on cell migration. The semaphorin–plexin system regulates actin cytoskeletal rearrangements and inhibits BMM migration by abrogating activation of RhoA but not Rac [16]. However, an earlier review study suggested that semaphorin function relies on Rac activity to potentially mediate actin rearrangement, membrane transport and endocytosis. It has also been shown that Sema3a plays a crucial role in regulating endothelial cell migration and angiogenesis, morphogenesis and leukocyte migration [41]. Sema3a may inhibit cytoskeleton rearrangement, which is required for cell migration and phagocytosis and for forming polarized cellular protrusions via actin polymerization and new transient adhesive structures [35, 41]; and our research is in agreement with these studies. Previous research also shows that Sema3a induces cell collapse and apoptosis in human macrophages resistant to Fas-induced apoptosis, indicating that Sema3a may act as an alternative apoptosis-inducing agent in conditions refractory to Fas-mediated apoptosis [26, 35, 41]. However, Sema3a showed no effect on Raw264.7 cell apoptosis under 0Gy radiation. The differences in *P53* gene expression reached statistical significance on the 3rd day 2Gy radiation in these experiments. In the study by Luca, monocytes cultured with M-CSF for 2 days showed a higher expression of *NRP-1* and *Plexin A1* in comparison with freshly isolated monocytes, and Sema3a caused a significant induction of apoptosis in the cells. This result indicates that Sema3a may induce monocyte apoptosis and work with M-CSF [26, 41]. However, we only applied Rankl without M-CSF in the present study to induce osteoclast differentiation under 0Gy radiation.

## Conclusion

Gamma radiation at 2Gy stimulated osteoclast differentiation of Raw264.7 cells and enhanced osteoclastic activity by inducing the production of ROS and inflammatory cytokines. Sema3a significantly reduced this inflammatory response and negatively regulated osteoclast differentiation and bone resorption activities. Sema3a also had an inhibitory effect on cell migration and promoted cell apoptosis after irradiation. All of these findings indicate that Sema3a may be a potential therapeutic target for radiation osteoporosis and other bone-related diseases.

## Supporting information

S1 Table The raw data of cell proliferation. (XLSX)

S2 Table The raw data of the number of osteoclast, the number of osteoclast nuclei and the raw data of the number of osteoclast nuclei. (XLSX)

S3 Table The raw data of the number of cell migration. (XLSX)

S4 Table The raw data of bone resorption area. (XLSX)

S5 Table The raw data of cell apoptosis. (XLSX)

S6 Table The raw data of ROS generation. (XLSX)

S7 Table The raw data of RT-PCR. (XLSX)
